# A Randomized Controlled Trial of Interleukin-1 Receptor Antagonist in a Rabbit Model of Ascending Infection in Pregnancy

**DOI:** 10.1155/S1064744901000382

**Published:** 2001

**Authors:** Robert S. McDuffie, Jill K. Davies, Kimberly K. Leslie, Scott Lee, Michael P. Sherman, Ronald S. Gibbs

**Affiliations:** 1 Department of Obstetrics and Gynecology Kaiser Permanente 2045 Franklin Street Denver CO 80205 USA; 2 University of Colorado Health Sciences Center Denver CO USA; 3 Department of Pediatrics University of California-Davis Davis CA USA

## Abstract

*Objective:* To determinewhether treatment with interleukin-1 receptor antagonist (IL1-ra) would affect amniotic fluid concentrations of tumor necrosis factor alpha (TNF-α) and prostaglandins or clinical or microbiological outcomes in a model of ascending bacterial infection in pregnancy.

*Methods:* Timed pregnant New Zealand white rabbits at 70% of gestation underwent endoscopic inoculation of the cervices with 10^6^–10^6^ *cfu Escherichia coli* . Animals were randomly assigned in a blinded manner to a 5-h intravenous infusion of human IL1-ra (10 mg/kg) or placebo beginning 1 – 2 h after inoculation. Blood was drawn fromthe does for assay of serum IL1-ra concentration before inoculation, at mid-infusion, after the infusion ended and at necropsy. At necropsy, temperature and cultures were taken, and aspirated amniotic fluid was pooled for assays of TNF-α, prostaglandin E_2_ (PGE_2_) and IL1-ra.

*Results:* Serum IL1-ra concentrations rose to a mean of 2 mg/ml at mid-infusion and fell markedly after the infusion to concentrations barely detectable at necropsy. Between the two groups, there were no significant differences in the rates of fever or positive cultures or in amniotic fluid concentrations of  PGE_2_ or TNF-α.One unique finding was the demonstration that administration of human IL1-ra to the does resulted inmeasurable concentrationsof human IL1-ra in the amniotic fluid.

*Conclusions:* Treatment with an intravenous infusion of human IL1-ra after cervical inoculation with *E. coli* did not affect clinical or microbiological outcomes or amniotic fluid concentrations of TNF-α or PGE_2_. This experiment provides the first demonstration of passageof human IL1-ra from the maternal bloodstreamto the amniotic fluid.


*Infect Dis Obstet Gynecol 2001;9:233–237*

### A randomized controlled trial of interleukin-1 receptor antagonist in a rabbit model of ascending infection in pregnancy

**Robert S. McDuffie Jr.[1], Jill K. Davies[2], Kimberly K. Leslie[2], Scott Lee[2], Michael P. Sherman[3] and Ronald S. Gibbs[2]**

[1]*Department of Obstetrics and Gynecology, Kaiser Permanente, Denver, CO*
[2]*University of Colorado Health Sciences Center, Denver, CO*
[3]*Department of Pediatrics, University of California-Davis, Davis, CA*

***Objective:*** To determine whether treatment with interleukin-1 receptor antagonist (IL1-ra) would affect amniotic fluid concentrations of tumor necrosis factor alpha (TNF-α) and prostaglandins or clinical or microbiological outcomes in a model of ascending bacterial infection in pregnancy.

***Methods:*** Timed pregnant New Zealand white rabbits at 70% of gestation underwent endoscopic inoculation of the cervices with $10^6$–$10^7$ cfu *Escherichia coli*. Animals were randomly assigned in a blinded manner to a 5-h intravenous infusion of human IL1-ra (10 mg/kg) or placebo beginning 1–2 h after inoculation. Blood was drawn from the does for assay of serum IL1-ra concentration before inoculation, at mid-infusion, after the infusion ended and at necropsy. At necropsy, temperature and cultures were taken, and aspirated amniotic fluid was pooled for assays of TNF-α, prostaglandin $E_2$ ($PGE_2$) and IL1-ra.

***Results:*** Serum IL1-ra concentrations rose to a mean of 2 μg/ml at mid-infusion and fell markedly after the infusion to concentrations barely detectable at necropsy. Between the two groups, there were no significant differences in the rates of fever or positive cultures or in amniotic fluid concentrations of $PGE_2$ or TNF-α. One unique finding was the demonstration that administration of human IL1-ra to the does resulted in measurable concentrations of human IL1-ra in the amniotic fluid.

***Conclusions:*** Treatment with an intravenous infusion of human IL1-ra after cervical inoculation with *E. coli* did not affect clinical or microbiological outcomes or amniotic fluid concentrations of TNF-α or $PGE_2$. This experiment provides the first demonstration of passage of human IL1-ra from the maternal bloodstream to the amniotic fluid.

Key words: ANIMAL MODEL; CYTOKINE; PROSTAGLANDIN; INTRA-AMNIOTIC INFECTION

Preterm birth and its sequelae remain the leading perinatal problem in the developed world. Clinically evident infection such as pyelonephritis or chorioamnionitis has long been associated with preterm birth. More recently, research has indicated that subclinical infection is an important cause of preterm birth perhaps accounting for up to 20–40% of cases[1]. Further, exposure of the preterm fetus to bacteria in the intrauterine environment has been related to adverse neonatal and childhood outcomes, especially cerebral palsy[2]. An overexuberant cytokine cascade may be an

Supported by grants from the March of Dimes (#6-FY97-0297) and the National Institutes of Health (HL52079).
Correspondence to: Robert S. McDuffie Jr., MD, Department of Obstetrics and Gynecology, Kaiser Permanente, 2045 Franklin Street, Denver, CO 80205, USA

important link between infection, fetal damage and neonatal disease[3,4]. Our group has recently provided evidence supporting the hypothesis that an excess of pro-inflammatory cytokines may cause fetal damage and neonatal and infant disease[5].

In our model of ascending clinical intra-amniotic infection in the pregnant rabbit, we have observed that after cervical inoculation with *Escherichia coli*, marked increases in amniotic fluid pro-inflammatory cytokines, tumor necrosis factor-α (TNF-α) and interleukin-1 (IL-1) α and β occur as early as 12–16 h after inoculation[6]. Increases in these cytokines are accompanied by increases in prostaglandins $E_2$ ($PGE_2$) and $F_{2\alpha}$ over the same time interval. We have also demonstrated that by 16 h after cervical inoculation with *E. coli*, there is placental expression and activation of nuclear factor-Kappa B (NF-κB), the principal transcriptional factor that upregulates pro-inflammatory cytokines[5]. In the same experiment, we found that the anti-inflammatory cytokine IL-1 receptor antagonist (IL-1ra) is present in the placenta and uterus, but does not increase in response to infection over the same 16-h interval.

This imbalance in the production of pro-inflammatory cytokines in response to infection led us to speculate on the use of IL-1ra as an intervention in this model. IL-1ra blocks the effect of IL-1 at the cell surface and thus downregulates pro-inflammatory activity, which in turn results in reduced production of TNF-α and prostaglandins. Further, animal models have demonstrated beneficial effects of IL-1ra infusions on hemodynamics after bacterial challenge[7–9]. In the present study, we conducted a randomized placebo-controlled trial to determine whether IL-1ra would affect the amniotic fluid concentrations of TNF-α and prostaglandins or clinical or microbiological outcomes in our model.

### SUBJECTS AND METHODS

This protocol was approved by the Animal Care Committee at the University of Colorado Health Sciences Center. Timed pregnant New Zealand white rabbits obtained from an approved vendor (Myrtle's Rabbitry, Thompson Station, TN) were anesthetized on day 21 or 22 of a 30-day gestation (70% of gestation). Animals were anesthetized with intramuscular xylazine (5 mg/kg) and ketamine (20 mg/kg). After external preparation with povodone iodine, an endoscope (Storz 27027B, Karl Storz Endoscopy America Inc., Culver City, CA) was inserted into the vagina to the cervix, and a sterile polyethylene cannula was advanced into the cervix under direct visualization. Through the cannula, 0.2 ml inoculum containing $10^{6-7}$ cfu of *E. coli* was injected into each cervix. *E. coli* (ATCC 12014) was aerobically inoculated into thioglycollate broth and grown for 8 h at 36°C before concentration and washing. After centrifugation the pellet was re-suspended in sterile phosphate buffer and re-centrifuged three times. A final suspension was then performed to achieve the final concentration. The final concentration was plated quantitatively before inoculation to confirm colony count, and an aliquot remaining in each syringe was plated after inoculation to confirm viability.

Animals were randomly assigned in a blinded manner to a 5-h intravenous infusion of IL-1ra in a dose of 10 mg/kg (kindly provided by Amgen Inc., Boulder, CO) or sterile saline (placebo). Human IL-1ra was used because a rabbit form was not available. After inoculation, animals were observed for up to 16 h. Blood was drawn from the does for assay of serum IL-1ra concentration before inoculation, at mid-infusion (2–3 h after the infusion began), after the infusion ended (8–10 h after the infusion began) and at necropsy.

At necropsy, a rectal temperature was taken. Cultures were taken from the uterus, amniotic fluid, peritoneum and blood of the doe by plating onto MacConkey and 5% sheep blood agar and by inoculation into thioglycollate broth. Pooled amniotic fluid was aspirated for assay of cytokines and prostaglandins.

TNF-α bioactivity was quantified by one of the authors (M.P.S.) with an established bioassay using a mouse fibrosarcoma cell line[5]. Assays of serum and amniotic fluid for human IL-1ra were performed by an enzyme-linked immunosorbent assay (ELISA) (kindly performed by Amgen Inc., Boulder, CO). $PGE_2$ was assayed by a competitive ELISA (reagents from Cayman Chemical, Ann Arbor, MI) that uses a monoclonal antibody to detect $PGE_2$. For this assay, the samples were diluted in assay buffer and quantified without purification. All assays were performed without knowledge of the assigned treatment group.

The sample size was calculated based on the outcome of fever defined as a rectal temperature of > 104°F as used in previous experiments in this model. We anticipated that 60% of untreated animals would have fever as would 10% of the treated animals. With alpha = 0.05 and beta = 0.2 and a 2 : 1 treatment to placebo ratio, 26 animals were needed in the treatment group and 13 animals in the control group. Categoric data were analyzed by the Fisher exact test. Continuous data were analyzed by the Wilcoxon rank sum test, since assumptions were not met. A *p* value of < 0.05 was considered to be significant.

### RESULTS

26 animals were studied in the treatment group and 13 animals in the control group. One doe died during the IL-1ra infusion. This animal was excluded from the analysis of fever at necropsy. No animal delivered prior to completion of the experiment at 16 h after inoculation. The serum concentrations of IL-1ra in the blood of the does at various time points are depicted in Figure 1. Concentrations rose quickly to a peak at mid-infusion, fell markedly after the infusion and were detectable in only small amounts at necropsy (16 h after inoculation). None of the placebo group had detectable concentrations of IL-1ra.

Outcomes are presented in Table 1. Between the two groups, there were no significant differences in the rates of fever or positive cultures, or in amniotic fluid concentrations of $PGE_2$ and TNF-α at necropsy. *E. coli* was the only organism isolated from any of the cultures. Amniotic fluid concentrations of IL-1ra at necropsy were detected only in treated animals. The concentrations found were similar to those in the maternal serum at necropsy (range 13.13–86.3 ng/ml). We did not collect amniotic fluid earlier in the experiment.

### DISCUSSION

In the rabbit model of intra-amniotic infection, treatment with an intravenous infusion of human IL-1ra after cervical inoculation with *E. coli* did not affect clinical or microbiological outcomes or amniotic fluid concentrations of TNF-α or $PGE_2$. We have documented the maternal serum concentrations of IL-1ra achieved during and after a 5-h infusion at a dose of 10 mg/kg. We have also demonstrated that this intravenous infusion of human IL-1ra into the pregnant rabbit led to detectable amniotic fluid concentrations of IL-1ra.

While these experimental conditions demonstrated no improvement in outcomes, it would be premature to conclude that IL-1ra is not a potential

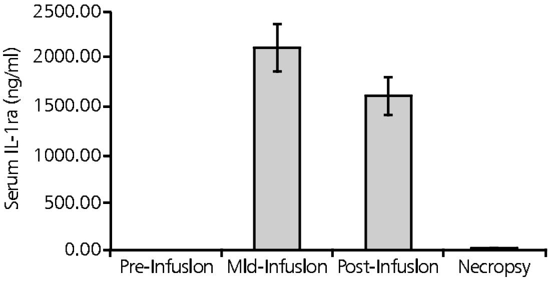

**Figure 1** Serum concentrations of IL-1ra in pregnant rabbits in relation to a 5-h infusion of human IL-1ra at a dose of 10 mg/kg. Intervals depicted are pre-infusion, mid-infusion (2–3 h after infusion began), post-infusion (8–10 h after infusion began) and necropsy

**Table 1** Outcomes at necropsy in pregnant rabbits inoculated cervically with *Escherichia coli* by treatment group

| | *IL-1ra* | *Placebo* | p *Value* |
|---|---|---|---|
| Fever | 17/25 (68%) | 8/13 (62%) | NS |
| Positive culture of uterus or amniotic fluid | 21/26 (81%) | 9/13 (69%) | NS |
| Any positive culture | 21/26 (81%) | 10/13 (77%) | NS |
| Amniotic fluid $PGF_2$ (ng/ml) | 2.98 (0.74SEM) | 3.55 (0.98SEM) | NS |
| Amniotic fluid TNF-α (pg/ml) | 259.9 (56.8SEM) | 296.7 (82.0SEM) | NS |
| Amniotic fluid IL-1ra (ng/ml) | 14.8 (4.2SEM) | ND | |

IL-1ra, interleukin-1 receptor antagonist; $PGE_2$, prostaglandin $E_2$; TNF-α, tumor necrosis factor alpha; NS, not significant; ND, none detected; SEM, standard error at the mean therapy. First, the dose of IL-1ra was chosen based on previous work in non-pregnant rabbit models. It is possible that the concentrations measured in the serum and amniotic fluid were inadequate to achieve a clinical effect. The serum concentrations of IL-1ra we measured at mid-infusion were much lower than those of Aiura and colleagues[8]: our mid-infusion concentration was 2 μg/ml compared with 10 μg/ml in that study. Our assay measured only human IL-1ra; thus, we cannot comment on endogenous production of rabbit IL-1ra. Also, we were unable to measure IL-1 in serum or amniotic fluid as there currently is no assay available for measuring this cytokine in the rabbit. Thus, we cannot comment on excess IL-1ra relative to IL-1. Second, we assessed endpoints only at necropsy after the infusion had ended. It is possible that there may have been benefits of IL-1ra infusion earlier in the course, but that ongoing infection reversed these by the end of the experiment. Third, it is possible that the human IL-1ra, though having a homology of approximately 75% with that of the rabbit, may not have been effective in this model. Fourth, an inoculum size of $10^{6-7}$ cfu in each horn may have produced so overwhelming a challenge that an effect of IL-1ra could not be demonstrated. Fifth, we administered IL-1ra intravenously into the maternal compartment, coinciding with the compartment where the *E. coli* inoculum was made. Nevertheless, it is possible that administering IL-1ra intra-amniotically (perhaps in combination with intravenous administration) might have had an effect. Finally, because we blocked only one pathway of inflammation, it is possible that other inflammatory cytokines such as TNF-α or IL-6 may have contributed to the findings. These points all provide possibilities for manipulation in future experiments.

We believe additional work with IL-1ra should be conducted because previous reports supported the use of IL-1ra to attenuate the effects of sepsis in animal models, especially the rabbit. Wakabayashi and colleagues[7] developed a model of sepsis in which rabbits were infused with heat-killed *E. coli* to produce shock. These authors demonstrated that intravenous infusion of 10 mg/kg bolus of IL-1ra followed by 15 μg/kg per min over 4 h reduced hypotension and associated leukopenia. Aiura and colleagues[8] infused rabbits with heat-killed *Staphylococcus epidermidis* and then treated rabbits with an intravenous infusion of IL-1ra 10 mg/kg bolus followed by 30 μg/kg per min over 4 h. Again, IL-1ra blocked a fall in mean arterial pressure after bacterial infusion compared with saline-infused controls. Finally, Vallette and colleagues[9] infused live group B streptococci into newborn piglets and treated them with an intravenous infusion of IL-1ra 40 mg/kg bolus followed by continuous infusion of 60 μg/kg per min. IL-1ra treated animals had significantly higher mean arterial blood pressures over time compared with controls. Because of these successes reported by others, we believe IL-1ra should be evaluated in additional experimental conditions as an adjunctive therapy to improve outcome in infection-induced preterm birth.

### REFERENCES

1. Gibbs RS, Romero R, Hillier SL, *et al.* A review of premature birth and sub clinical infection. *Am J Obstet Gynecol* 1992;116:1515–28
2. Bejar R, Wozniak P, Allard M, *et al.* Antenatal origin of neurologic damage in newborn infants. I. Preterm infants. *Am J Obstet Gynecol* 1988;159: 357–63
3. Leviton A. Preterm birth and cerebral palsy: is tumor necropsy factor the missing link? *Dev Med Child Neurol* 1993;35:553–8
4. Yoon BH, Romero R, Yang SH, *et al.* Interleukin-6 concentrations in umbilical cord plasma are elevated in neonates with white matter lesions associated with periventricular leukomalacia. *Am J Obstet Gynecol* 1996;174:1433–40
5. Leslie KK, Lee SL, Woodcock SM, *et al.* Acute intrauterine infection results in an imbalance between pro- and anti-inflammatory cytokines in the pregnant rabbit. *Am J Reprod Immunol* 2000;43: 305–11
6. McDuffie RS, Sherman MP, Gibbs RS. Amniotic fluid tumor necrosis factor-α and interleukin-1 in a rabbit model of bacterially induced preterm pregnancy loss. *Am J Obstet Gynecol* 1992;167:1583–8
7. Wakabayashi G, Gelfand JA, Burke JF, *et al.* A specific receptor antagonist for interleukin 1

prevents *Escherichia coli*-induced shock in rabbits. *FASEB J* 1991;5:338–43

8. Aiura K, Gelfand JA, Burke JF, *et al.* Interleukin-1 (IL-1) receptor antagonist prevents *Staphylococcus epidermidis*-induced hypotension and reduces circulating levels of tumor necrosis factor and IL-1β in rabbits. *Infect Immun* 1993;66:3342–50
9. Vallette JD, Goldberg RN, Suguihara C, *et al.* Effect of an interleukin-1 receptor antagonist on the hemodynamic manifestations of group B streptococcal sepsis. *Pediatr Res* 1995;38:704–8

RECEIVED 03/08/01; ACCEPTED 08/03/01
